# Non‐Hodgkin lymphoma in a kidney transplanted patient with methylmalonic acidemia: Metabolic susceptibility and the role of immunosuppression

**DOI:** 10.1002/jmd2.12411

**Published:** 2024-01-10

**Authors:** Alberto B. Burlina, Alessandro P. Burlina, Renzo Mignani, Chiara Cazzorla, Daniela Gueraldi, Andrea Puma, Christian Loro, Matthias R. Baumgartner, Vincenza Gragnaniello

**Affiliations:** ^1^ Division of Inherited Metabolic Diseases, Department of Women's and Children's Health University Hospital of Padua Padua Italy; ^2^ Division of Inherited Metabolic Diseases, Department of Women's and Children's Health University of Padua Padua Italy; ^3^ Neurology Unit St Bassiano Hospital Bassano del Grappa Italy; ^4^ Nephrology and Dialysis Department Infermi Hospital Rimini Italy; ^5^ Division of Metabolism and Children's Research Center University Children's Hospital Zurich, University of Zurich Zurich Switzerland

**Keywords:** chemotherapy toxicity, immunosuppression, kidney transplantation, methylmalonic acidemia, mitochondrial dysfunction, post‐transplant lymphoproliferative disorders

## Abstract

Methylmalonic acidemia cblB type (MMA cblB) is an autosomal recessive inborn error of amino acid metabolism that results in impaired synthesis of adenosylcobalamin, a cofactor of methylmalonyl‐CoA mutase. It presents with episodes of coma, vomiting, hypotonia, metabolic acidosis, and hyperammonemia. End‐stage kidney disease is a long‐term complication. Treatments include vitamin B12 supplementation, L‐carnitine, and a low‐protein diet. Liver, kidney, or combined liver‐kidney transplantations are promising options, but they are not without complications. We report a patient suffering from MMA cblB who developed end‐stage kidney disease at 18 years of age. Kidney transplantation allowed him to recover normal kidney function and good metabolic control. Unfortunately, after two decades, he developed non‐Hodgkin lymphoma and severe chemotherapy toxicity which led to his death. The risk of lymphoproliferative diseases is known to increase after solid organ transplantation. However, in MMA, factors including mitochondrial dysfunction and oncometabolites, may further increase the risk of malignancy and drug toxicity. Our report highlights the importance of considering the increased risk of cancer in long‐term follow‐up of MMA cblB patients, especially after solid organ transplantation. Moreover, when chemotherapy is needed, the increased risk of toxicity and metabolic decompensation should be considered and monitored.

**Synopsys**

Patients with methylmalonic acidemia have an increased risk of cancer, especially after solid organ transplantation, and increased susceptibility to chemotherapy toxicity.

## 1 INTRODUCTION

Methylmalonic acidemia cblB type (MMA cblB, OMIM 251110) is an autosomal recessive disease, due to variants in the *MMAB* gene (OMIM 607568) encoding the mitochondrial enzyme ATP:cobalamin adenosyltransferase, which converts cobalamin to adenosylcobalamin using ATP. Adenosylcobalamin is cofactor for methylmalonyl‐CoA mutase (MCM), which converts L‐methylmalonyl‐CoA into succinyl‐CoA that then enters the tricarboxylic acid cycle.^1^, ^2^

MMA cblB can present as early‐onset acute neonatal decompensation or as late‐onset symptoms including failure to thrive, anorexia, vomiting, and developmental delay.^3^, ^4^ The disease is included in the newborn screening panel in many countries, including Italy.^5^, ^6^, ^7^

Management includes vitamin B_12_ supplementation (only for responsive patients), a low‐protein high‐energy diet, L‐carnitine supplementation, N‐carbamylglutamate, and antibiotics used intermittently to reduce propionate production by the gut flora.^3^, ^8^, ^9^, ^10^

Despite treatment, these patients can develop a variety of long‐term complications, including progressive renal disease (tubulointerstitial nephritis due to secondary mitochondrial disease), which commonly reaches end‐stage kidney disease during childhood or adolescence.^11^, ^12^ Liver, kidney, or combined liver‐kidney transplantations are considered an alternative to medical treatment. Kidney transplantation corrects renal failure and provides partial enzymatic activity.^13^, ^14^, ^15^

A previous study by Brassier et al. demonstrated that kidney transplantation decreased urinary and plasma methylmalonic acid levels, reducing the number of metabolic decompensations and increasing the dietary protein allowance. However, the risk of metabolic decompensation is not entirely suppressed, presumably because enzymatic activity is insufficient in case of intercurrent diseases.^16^ Moreover, these procedures are not without danger and entail severe challenges, such as the need for prolonged immunosuppression. Post‐transplant lymphoproliferative disorders (PTLD) represent one of the most feared and fatal complications following kidney transplantation, occurring in about 1% of patients.^17^ Recent studies have evaluated specific short‐term complications of post‐transplantation immunosuppression in MMA patients, including acute/subacute neurological complications such as posterior reversible encephalopathy syndrome (PRES)^18^; meanwhile, less is known about long‐term risks, such as malignancies. Furthermore, little is known about the management of malignancies in MMA patients and chemotherapy toxicity in the context of mitochondrial impairment and increased cellular oxidative stress.

We report the case of a patient with cblB MMA who developed non‐Hodgkin lymphoma more than 20 years after received a kidney transplantation, and experienced severe chemotherapy toxicity that led to his death.

## 2 CASE REPORT

The patient, an Italian male born in 1983 from non‐consanguineous parents, was diagnosed with MMA at the age of 1 month after an episode of severe ketoacidosis. Urinary organic acid analysis showed increased level of methylmalonic and methylcitric acids. Molecular examination revealed a homozygous variant of the *MMAB* gene (c.556C>T, p.R186W—courtesy of Professor MR Baumgartner, Zurich), confirming the diagnosis of cblB MMA. Both parents were carriers of this variant.

Despite the low‐protein diet (1–1.5 g/kg/day) and pharmacological therapy with L‐carnitine (100 mg/kg/die), he developed progressive kidney failure and required peritoneal dialysis at the age of 18 years. After 6 months of dialysis, he received a kidney transplantation from a deceased donor, with normalization of renal function (creatinine 1 mg/dL, eGFR according to Cockcroft‐Gault: 118.6 mL/m^2^). He was immunosuppressed with basiliximab, followed by steroids (withdrawn 1 year after transplantation), tacrolimus bid, and mycophenolate mofetil, modulated over time based on therapeutic drug monitoring and the health of the transplanted organ (tacrolimus 2.5–3 mg/day with serum levels ranging from 5 to 7 ng/mL, mycophenolate mofetil 500–750 mg/day). The immediate post‐transplantation course was complicated by two episodes of syncope followed by seizures. The patient was admitted to a local hospital and underwent an (unremarkable) CT scan, before being transferred to our center. Neurological examination at admission was normal. Brain MRI showed bilateral lesions in the tegmentum and pons, which were present also in diffusion weighted imaging, and were consistent with recent stroke‐like lesions. Antiepileptic treatment with oxcarbazepine resolved seizures, and was suspended after 10 years of treatment. Several episodes of tonsillitis occurred 5 years after transplantation, and the infection was resolved with radiofrequency treatments.

**FIGURE 1:**
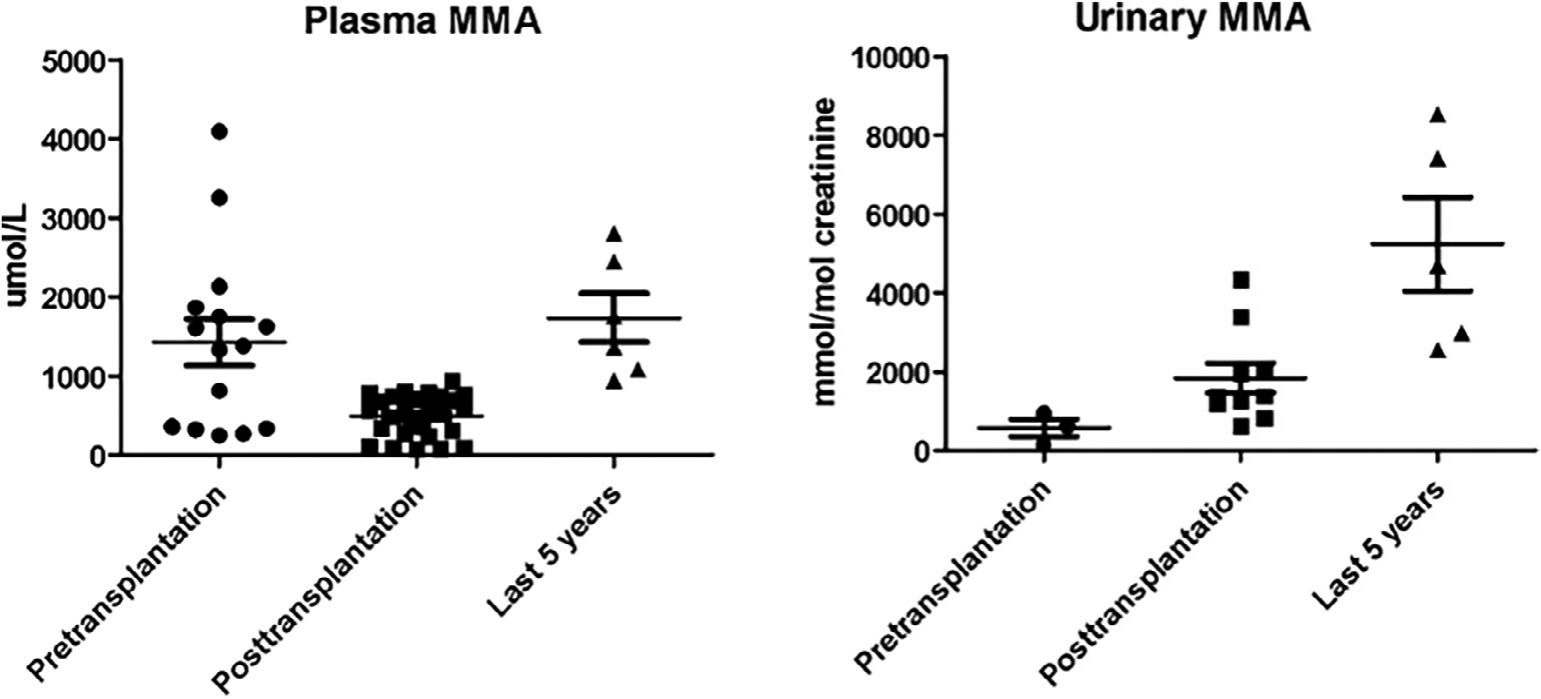
Plasma and urinary methylmalonic acid levels pre‐kidney transplantation and during short‐ and long‐term follow up. Plasma methylmalonic was greatly reduced after kidney transplantation, while urinary methylmalonic increased due to improved kidney function. Both parameters worsened during long‐term follow‐up.

The patient continued a low protein diet (45 g/day) and carnitine supplementation (100 mg/kg/die) for management of the metabolic disease. Metabolic parameters improved greatly after transplantation, with reduced plasma MMA and increased urinary excretion (Table 1, Figure 1). The patient did not experience episodes of acute decompensation. He had normal academic achievement, autonomy, and socio‐professional integration. He earned a degree in biology and was employed as laboratory assistant.

**TABLE 1:** Clinic and metabolic parameters over time.

	Protein intake	Therapy	Kidney function	Plasma MMA μmol/l (nv 0–2)^a^	Urine MMA mmol/mol crea (nv 0.1–5)^a^	Clinical features/complications
6 months pre‐kidney transplantation	30 g/day	L‐carnitine 100 mg/kg	eGFR <15 mL/m^2^	1426.2 (244.5–4094.8)	564.7 (145.2–934.3)	End stage renal disease
6 months post‐kidney transplantation	45 g/day	L‐carnitine 100 mg/kg, steroid, tacrolimus, mycophenolate mofetil	eGFR 118.6 mL/m^2^	488.31 (67–93.3)	1830.4 (613.2–4332.9)	Seizures (tacrolimus toxicity)
Last 5 years	45 g/day	L‐carnitine 100 mg/kg, tacrolimus, mycophenolate mofetil	eGFR 44.6 mL/m^2^	1806.68 (942.2–2802.7)	5234.8 (2549.5–8529)	Progressive kidney disease, acidosis
Last 6 months	45 g/day	L‐carnitine 100 mg/kg, tacrolimus, R‐CHOP	eGFR 21.4 mL/m^2^	3549.8	1830.3	Non‐Hodgkin lymphoma, metabolic decompensation

Abbreviation: MMA, methylmalonic acid.

^a^ Mean and range.

After 10 years, kidney function progressively worsened (creatinine reached 2–2.2 mg/dL, eGFR 49.1–44.6 mL/m^2^, without proteinuria at the urine analysis); a renal biopsy was not performed. The patient had chronic acidosis (HCO_3_^−^ 15.9–18.2 mEq/L, nv 22.0–26.0; Base Excess – 12.2/−6.8 mEq/L, nv −5/+5) that required sodium bicarbonate supplementation (7.5 g/day in three doses). Metabolic parameters worsened (Table 1, Figure 1), with increase of plasma and urinary MMA, but he never developed acute ketoacidosis episodes. He continued immunosuppression with tacrolimus (serum level maintained between 5 and 7 ng/mL) and mycophenolate mofetil. In the same period, he had a chicken pox infection complicated by kerato‐uveitis of the right eye with severe decrease of *visus*. Treatment with oral valacyclovir led to progressive but partial restoration of *visus*; immunosuppressive therapy was unmodified.

At age 39, during routine follow‐up, abdominal ultrasound revealed a mass in the cecal region. Endoscopy revealed concentric stenosis with ulcers in the ascending colon. Histological examination revealed centrogerminative diffuse large B cell lymphoma (DLBCL). Immunohistochemistry was negative for bcl2 and myc. Immunostaining was negative for Human herpes virus 8 and in situ hybridization was negative for Epstein–Barr virus (EBV) RNA. Staging tests were performed. Abdominal CT scan showed a concentric parietal mass of the ascending colon, measuring 16 × 10 cm^2^ and involving the mesentery, with intense ^18^F‐FDG accumulation on PET (SUV max 18.38). There was no bone marrow or central nervous system involvement. Advanced stage (IV E gut IPI2) DLBCL was diagnosed. Serum E‐Barr virus DNA was negative as well. The patient was followed at a local hospital.

After diagnosis, mycophenolate mofetil was immediately discontinued and after 1 month the patient started chemotherapy according to the R‐CHOP protocol (2 cycles of rituximab +6 cycles of cyclophosphamide, vincristine, and doxorubicin). Urinary MMA was unchanged. After the second cycle, the patient developed severe pancytopenia and kidney failure (creatinine 4.58 mg/dL, eGFR 21.4 mL/m^2^), and was treated with transfusions of platelets and red blood cells and IV hydration. At the third cycle, the dosage of chemotherapy drugs was reduced (75%) but the patient presented again pancytopenia, fever, and kidney failure, treated with transfusions, antibiotics, and IV hydration. He also developed sensory neuropathy in the hands and feet attributed to vincristine. Despite the toxicity, restaging with PET‐TC scan showed marked both dimensional and metabolic response of the abdominal lesion (7 mm, SUV max 3.6). The IV cycle of chemotherapy was performed at reduced dosages.

After a few days, the patient presented confusion, dyspnea, and tachypnea. At admission, blood tests showed severe metabolic acidosis with high lactic acid (pH 6.91, nv 7.35–7.45; HCO_3_^−^ 3.7 mEq/L, nv 22.0–26.0; Base Excess −28.9 mEq/L, nv −5/+5; Anion gap 47.6 mEq/L, nv 8–16; Lac 23.39 mmol/L nv 0.8–2.0), and hyperglycemia (glucose 367 mg/dL), treated with insulin and hemodiafiltration, with normalization within 24 h. Brain CT scan revealed symmetrical hypodense lesions of the caudate nuclei, typical of MMA. Chest CT showed a left basal consolidation, that, associated with leukocytosis (49.840/mm^2^) and increased CRP (19 mg/dL), led to diagnosis of pneumonia. Due to immunodepression status, patient was treated with meropenem, linezolid, and liposomal amphotericin B.

Despite the therapy, after 2 days he developed cardiorespiratory insufficiency for which he was admitted to the intensive care unit and mechanically ventilated. After 10 days, the course was complicated by pulmonary embolism, treated with anticoagulation. Suspected hypoxic/ischemic brain lesions prompted a CT scan, which revealed massive hemorrhage in the left basal ganglia, with edema and the presence of the “swirl sign” (indicator of active bleeding and predictor of poor outcome); similar hemorrhagic lesions were present in the caudate and lenticular nuclei of the right basal ganglia; hydrocephalus with blood inside the left lateral ventricle, third and fourth ventricle; severe edema enhanced by the compressive effect of the hemorrhages. The patient died the next day, 4 months after being diagnosed with lymphoma. No additional data regarding MMA values were available.

## 3 DISCUSSION

We report a case of non‐Hodgkin lymphoma (NHL) in the long‐term follow‐up of a patient with cblB MMA, after kidney transplantation.

In addition to the highest risk of cancer in kidney transplanted patients, compared to the general population,^19^ several factors in patients with MMA may increase the risk of malignancy.

Case reports of liver and kidney cancer in MMA patients exist. Forny et al. described 5 cases of liver neoplasm in early onset MMA patients, including a cblB MMA patient. This latter required hemodialysis since 16 year‐old for stage 4 chronic kidney disease. After 6 years, she presented severe metabolic decompensation and passed away a few days later. A postmortem report confirmed hepatocellular carcinoma. Two other cases had chronic kidney disease before the development of liver tumors. One of them developed hepatoblastoma at the age of 11 years, 18 months after kidney transplantation. His neurological condition worsened, with bilateral pallidum lesions. During chemotherapy, plasma ammonia increased up to 150 μmol/L, as well as lactatemia (12.5 mmol/L) and glycemia. The patient died from toxicity within 3 weeks of initiating chemotherapy.^20^, ^21^, ^22^ The last two cases presented early development of hepatoblastoma (4 and 19 months of age). One of them experienced very pronounced toxicity from chemotherapy, with severe metabolic acidosis and hyperglycemia. Finally, Potter et al. described a renal cell carcinoma in a 6‐year‐old patient with methylmalonic aci*dem*ia (MUT gene) and stage 4 chronic kidney disease.^23^

These cases highlight some very important aspects in the management of patients with MMA: there may be a greater cancer risk, there seems to be an association with end‐stage renal disease (however, this may be linked to older age and more serious MMA disease in patients who develop cancer), and finally a greater susceptibility to the side effects of chemotherapy, including metabolic decompensation. Our patient also presented with these features. After kidney transplantation, his metabolic outcome was well‐controlled for 20 years. MMA levels increased progressively, but he did not experience episodes of metabolic decompensation requiring hospitalization. He followed a lacto‐vegetarian diet with a protein intake of 45 g/day. His kidney function was declining when he developed the cancer. Despite a good response to chemotherapy, he developed severe drug toxicity, comprising the most frequent side effects (cytopenia, fever, neuropathy), but also had a loss of metabolic balance with acidosis, and basal ganglia stroke which finally led to death. Unlike the previously reported cases above, he did not have a solid tumor, but a non‐Hodgkin lymphoma.

After solid organ transplantation, the risk of developing lymphoproliferative disease (PTLD) is higher than that of the general population due to prolonged immunosuppression.^24^, ^25^, ^26^ A recent review reports the follow‐up of 96 patients with MMA who underwent liver (*n* = 50), kidney (*n* = 8) or combined transplantation (*n* = 38) in the last 10 years. The median follow up was 2.5 years (0.2–12 years). Only 1 patient, a six‐year‐old MMA girl with *MUT* variants, developed a PTLD, occurring 1 year after liver‐kidney transplantation. She recovered with reduction of immunosuppression and rituximab, without requiring chemotherapy.^27^, ^28^ Conversely, in a long‐term follow‐up after liver transplantation of two cohorts of patients with propionic acidemia (12 and 14 patients, respectively), 4 developed PTLD (0.1–13.5 years after transplantation).^29^, ^30^ Longer follow‐up of transplanted MMA patients may reveal more cases of PTLD.

The occurrence of cancer in organic acidemias might be multifactorial. Mitochondrial dysfunction is a well‐recognized pathophysiological mechanism of MMA. Mitochondria are responsible for energy production but are also involved in cell proliferation and apoptosis (programmed cell death) and mitochondrial disorders have been associated with malignancies.^31^

Mitochondrial impairment increases the production of reactive oxygen species ROS, also because MMA inhibits the transport of glutathione in mitochondria via a dicarboxylate carrier, resulting in redox imbalance and mitochondrial antioxidant defense exhaustion.^32^ ROS plays a role in oncogenesis, causing DNA damage and activation of ROS‐dependent pro‐oncogenic signaling pathways.^22^

Moreover, oncometabolites increase neoplastic vulnerability via their effect on key enzymes regulating metabolic pathways that facilitate cell survival or dedifferentiation.^33^ Propionyl‐CoA is known to modify histone acetylation.^22^, ^34^ MMA induces SOX4 by activating autocrine TGFß signaling, resulting in transcriptional reprogramming of cancer cells that endows them with malignant properties such as invasiveness and metastatic potential. For example, MMA can induce a pro‐metastatic epithelial‐to‐mesenchymal transition‐like phenotype, with a decline in E‐cadherin and a concurrent increase in fibronectin and vimentin.^35^, ^36^

Several factors may increase the risk of PTLD. In early post‐transplantation onset PTLD, the induction therapy and the EBV infection are the most important. More than 50% of PTLD cases are EBV‐related, especially in case of EBV donor‐recipient mismatch.^17^ Indeed, the role of anti‐thymocyte globulin induction therapy as a risk factor for PTLD is controversial. However, our patient was EBV negative at the onset of PTLD and induction was accomplished with basiliximab. By contrast, in patients with late‐onset PTLD the most important risk factors are patient age and the immune status. In particular, calcineurin inhibitors like tacrolimus are associated with an increased risk of PTLD.^37^ Tacrolimus serum levels in our patient were consistently in the upper level of the target range, and may have resulted in excessive immunosuppression, as suggested by the documented infective episodes before PTLD onset. Therefore, the cumulative immunosuppression therapy may have increased the risk of PTLD.

Finally, our patient developed severe chemotherapy toxicity. Mitochondrial impairment can also explain the increased drug toxicity in MMA patients. Accordingly, our patient developed acidosis, high lactic acid, and basal ganglia stroke, secondary to mitochondrial impairment. This susceptibility should be considered in the chemotherapy plans of patients with organic acidemias.

In conclusion, our patient represents the first report of NHL in the long‐term follow‐up of a kidney transplanted patient with cblB‐MMA. Although prolonged post‐transplant immunosuppression is a risk factor for lymphoproliferative diseases, our patient presented lymphoma many years after the transplant, without a known infectious cause, and simultaneously with progressive renal and metabolic deterioration. Based on our experience, and previously described cases of cancer in patients with methylmalonic acidemia, we could speculate that oncogenesis could be the result of chemically induced damage stemming from the underlying metabolic disorder and longstanding kidney disease. More patients with long‐term follow‐up are necessary to appreciate the risk of malignancy in organic acidurias, especially after solid organ transplantation.

## FUNDING INFORMATION

This research received no external funding.

## CONFLICT OF INTEREST STATEMENT

The authors of this manuscript have no conflict of interest to disclose.

## INFORMED CONSENT STATEMENT

Informed consent was obtained from the parents of the patient.

## Data Availability

Data available on request due to privacy/ethical restrictions.
